# Estimating the short-term effect of PM_2.5_ on the mortality of cardiovascular diseases based on instrumental variables

**DOI:** 10.1186/s12889-024-18750-0

**Published:** 2024-08-01

**Authors:** Guiming Zhu, Le Zhao, Tao Lin, Xuefeng Yu, Hongwei Sun, Zhiguang Zhang, Tong Wang

**Affiliations:** 1 Department of Health Statistics, School of Public Health, Shanxi Medical University, Taiyuan, 030001 China; 2 MOE Key Laboratory of Coal Environmental Pathogenicity and Prevention, Taiyuan, 030001 China; 3 Binzhou Center for Disease Control and Prevention, No. 413, Huanghe 2nd Road, Binzhou, 256600 China; 4 School of Public Health and Management, Binzhou Medical University, Yantai, 264000 China

**Keywords:** PM_2.5_, Instrumental variables, Cardiovascular diseases, Short-term effect

## Abstract

**Background:**

PM_2.5_ can induce and aggravate the occurrence and development of cardiovascular diseases (CVDs). The objective of our study is to estimate the causal effect of PM_2.5_ on mortality rates associated with CVDs using the instrumental variables (IVs) method.

**Methods:**

We extracted daily meteorological, PM_2.5_ and CVDs death data from 2016 to 2020 in Binzhou. Subsequently, we employed the general additive model (GAM), two-stage predictor substitution (2SPS), and control function (CFN) to analyze the association between PM_2.5_ and daily CVDs mortality.

**Results:**

The 2SPS estimated the association between PM_2.5_ and daily CVDs mortality as 1.14% (95% CI: 1.04%, 1.14%) for every 10 µg/m^3^ increase in PM_2.5_. Meanwhile, the CFN estimated this association to be 1.05% (95% CI: 1.02%, 1.10%). The GAM estimated it as 0.85% (95% CI: 0.77%, 1.05%). PM_2.5_ also exhibited a statistically significant effect on the mortality rate of patients with ischaemic heart disease, myocardial infarction, or cerebrovascular accidents (*P* < 0.05). However, no significant association was observed between PM_2.5_ and hypertension.

**Conclusion:**

PM_2.5_ was significantly associated with daily CVDs deaths (excluding hypertension). The estimates from the IVs method were slightly higher than those from the GAM. Previous studies based on GAM may have underestimated the impact of PM_2.5_ on CVDs.

**Supplementary Information:**

The online version contains supplementary material available at 10.1186/s12889-024-18750-0.

## Introduction

Air pollution has seriously affected people’s health and has become an increasingly serious public health problem in China. With the continuous acceleration of urbanization in China, the problem of the urban atmospheric environment is becoming increasingly serious. PM_2.5_ is the main component of air pollution and is also a characteristic indicator for evaluating the relationship between air pollution and disease burden. To date, PM_2.5_ is still an important pollutant affecting the air quality in most regions of China. PM_2.5_ can induce and aggravate the occurrence of cardiovascular diseases (CVDs). A large number of epidemiological studies have shown that outdoor air pollution poses a serious threat to human health [1–5]. In different cities, when the concentration of air pollutants increases, the number of hospital visits and the number of deaths from CVDs increase to a certain extent. The mortality rate in cities with severe air pollution is significantly higher than that in less polluted cities. In addition, many toxicology and human exposure studies [6, 7] have shown that PM_2.5_ is associated with changes in blood pressure, inflammation, autonomic function, endothelial function, and thrombus formation. Among the various air pollutants in China, PM_2.5_ is the most serious, and it also poses a great threat to the CVDs of residents. Therefore, accurate estimation of the causal impact of PM_2.5_ on major CVDs is of great significance for further controlling air pollution emissions, formulating air quality standards, and improving residents’ health.

Compared to experimental research, one of the most prominent limitations in causal inference for observational studies is the need for effective management of confounders. However, the instrumental variables (IVs) method is not susceptible to all confounders, and its theory and application in both linear and nonlinear models have been extensively studied [8, 9]. Schwartz [10] was the first to apply the IVs method to estimate the acute impact of air pollution. Furthermore, there is limited research on applying the control function (CFN) method to the analysis of air pollution. Additionally, few studies in China utilize IVs method to estimate the short-term effects of air pollution. Therefore, our study aims to utilize two IVs methods to estimate the robust and reliable short-term effects of PM_2.5_ on the mortality of CVDs among residents in China.

## Materials and methods

### Study area

Binzhou, Shandong Province, is a city with severe ambient PM_2.5_ and a typical area where smog events frequently occur. Approximately 15,000 people die of CVDs every year, accounting for more than 50% of all deaths. The resident population is approximately 3.9 million, and the total area is 9,660 square km (http://tj.binzhou.gov.cn/), as shown in Supplemental Figure S1. Additionally, the flat terrain, relatively stable climate, and infrequent occurrence of extreme weather events, such as typhoons, are similar to the situation in most cities in China. It is regarded as an appropriate place to study the effects of PM_2.5_ exposure on mortality from CVDs.

### Exposure data

**Fig. 1:**
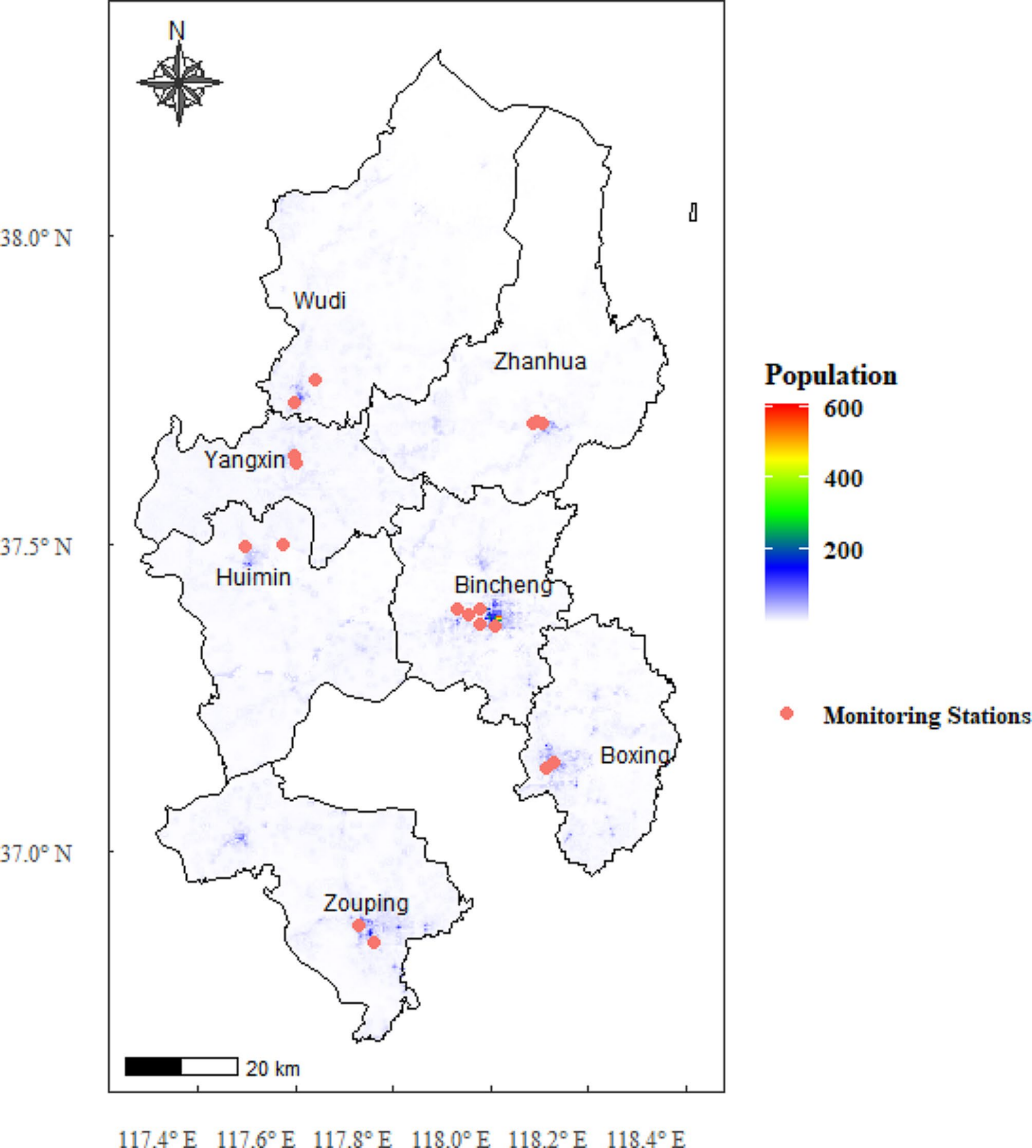
Air pollutant monitoring stations and population in Binzhou

Most studies on estimating the health effects of environmental pollution often directly use the monitoring data from environmental monitoring stations as individual exposure levels without considering the spatial heterogeneity of pollutants within cities (for example, in our study, most of the monitoring stations were located in areas with large populations, as shown in Fig. 1.). This may eventually lead to bias in health impact assessments. Taking into account computational efficiency and providing a visual representation of the impact of various factors on PM_2.5_, we use the land use regression (LUR) model [11] to estimate the spatial and temporal distributions of PM_2.5_ in Binzhou. We obtained PM_2.5_ data from air quality monitoring points as the dependent variable. Land use, traffic, industrial emissions, meteorology, terrain, population distribution, and other factors were used as independent variables (Supplemental Table S1 and Supplemental Table S2). Then, the longitude and latitude coordinates of the deceased were obtained according to their address before death. Because studies examining the acute association of PM_2.5_ with daily mortality commonly use similar 2-day means [10], we extracted PM_2.5_ within the day of death (lag 0) and the day before death (lag 1).

### Death data

The death data were obtained from the death registration report information system of the Binzhou Center for Disease Control and Prevention in Shandong Province includes the address of the deceased. The cause of death of the deceased was coded and classified according to the International Statistical Classification of Diseases and Related Health Problems 10th Revision (ICD-10), and the diseases included in this study were classified as CVDs (ICD-10 code: I00-I99) and major CVDs, including ischaemic heart disease (IHD, ICD-10 code: I20-25), cerebrovascular accident (CVA, ICD-10 code: I61, I63), myocardial infarction (MI, ICD-10 code: I21-22) and hypertension (HTN, ICD-10 code: I10-I15). Our study was reviewed by the Ethical Review Committee of the Binzhou Center for Disease Control and Prevention (Project No:202,301). Our study did not involve human experiments or the use of human tissue samples. All respondents and relevant personnel signed informed consent forms before the investigation.

### Instrumental variables

**Fig. 2:**
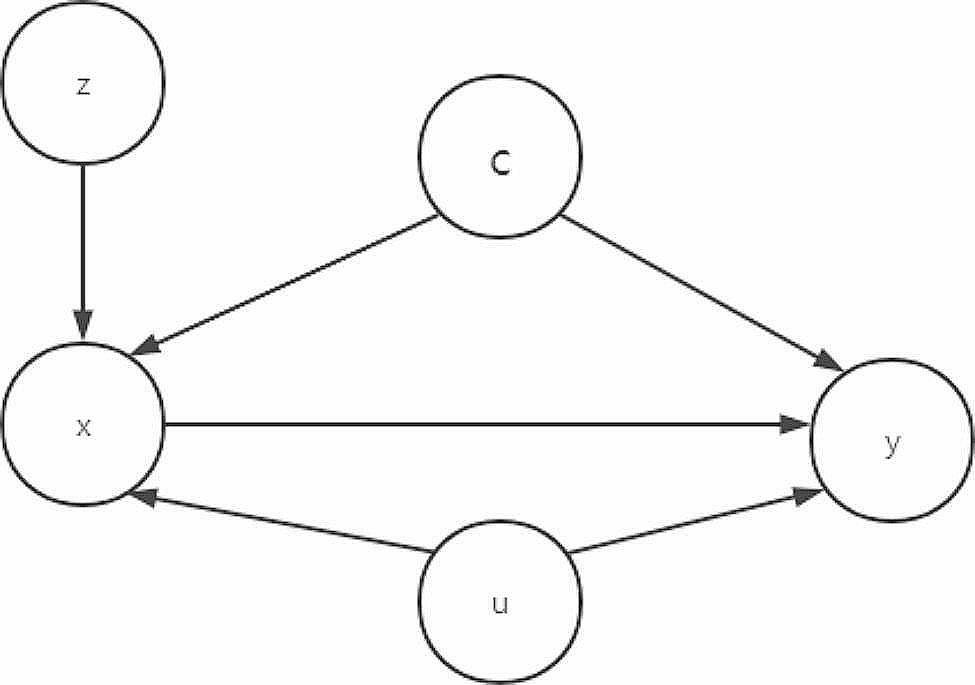
DAG with IVs (y is the outcome, *x* is the exposure, c represents the known confounders, u represents the unobserved confounders, and z is the IVs)

When addressing unobserved confounders, the IVs model emerges as a primary tool to mitigate these challenges. The IVs was first proposed by P.G. Wright [12] to circumvent the influence of unobserved confounders. However, the IVs model must satisfy the following three basic assumptions, as shown in Fig. 2:

1. Independence: z is independent of c and u;
2. Correlation: z is related to x;
3. Exclusive: given x and c, u, z and y are independent.

It can be proven that asymptotically unbiased estimation of the causal effect can be obtained under these basic assumptions of the IVs [13].

Previous studies on the estimation of the health effects of air pollution using IVs [10, 14, 15] have guided our selection of IVs in this study. We have chosen wind speed (WS) and boundary layer height (BLH) as IVs. Put simply, under certain pollutant conditions, the height of the boundary layer in the vertical direction correlates directly with the effective air volume for pollutant diffusion and dilution. A higher BLH implies a larger volume of pollutants that can be diluted, facilitating the vertical dispersion of pollutants and thereby reducing their concentration [16]. BLH is unlikely to be associated with daily mortality other than by affecting air pollution changes. Air pollutants emitted in local areas also exhibit characteristics of horizontal transport. The impact of local air pollution sources increases with decreasing WS and vice versa. Except for extreme events (such as typhoons), WS is unlikely to influence population mortality directly; rather, it is only air pollution that can affect population health. Changes in WS or BLH do not alter the behavior of the exposed population; for example, there is no association with other behaviors that affect short-term CVDs mortality (such as the number of cigarettes smoked, changes in daily diet, or alcohol consumption) [10].

However, PM_2.5_, WS and BLH may vary with time and temperature. Therefore, consistent with most previous literature [10, 14, 15], it is necessary to remove the influence of temperature and temporal trends. Specifically, first we fit the following model:

1 $$\begin{array}{l}{\varepsilon _1} = p{m_t} - ({\beta _0} + ns\left( {time,df} \right)\\+ ns\left( {te{m_t},df} \right) + dow)\end{array}$$

In formula (1), ${\beta }_{0}$ represents the intercept, *t* denotes time, $ns$ indicates the cubic natural spline, and $time$ is the time to control the influence of long-term trends.$df$ is the degree of freedom (obtained by cross-validation [17, 18]), here, the degrees of freedom for the time spline and temperature spline are 52 and 15, respectively. ${tem}_{t}$ represents the temperature at time *t*, $dow$ denotes the dummy variable for the day of the week to control the impact of short-term fluctuations, ${pm}_{t}$ is the PM_2.5_ at time t, and ${\epsilon }_{1}$ is the model residual.

**Fig. 3:**
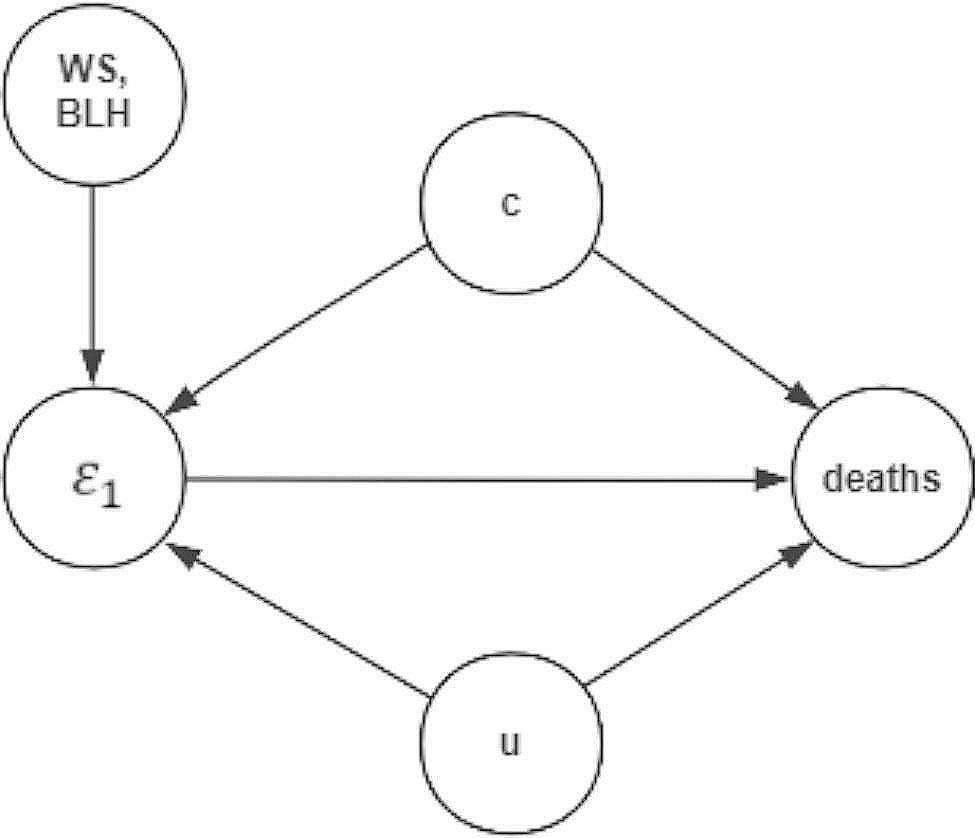
DAG with our study (deaths is our outcome, ${\epsilon }_{1}$ is the exposure, WS is wind speed, BLH is boundary layer height, c represents the known confounders (Such as temperature, etc.), u represents the unobserved confounders)

The ${\epsilon }_{1}$ is independent of the temporal trends, seasons and temperature. It represents a component of PM_2.5_ and comprises selected IVs and other factors. In this study ${\epsilon }_{1}$ is used as the exposure variable, as shown in Fig. 3:

### Statistical analysis

#### Descriptive analysis

The mean, standard deviation, median, and other common descriptive statistical analyses were carried out on the meteorological data, air pollutant data, and daily deaths. The correlation between meteorological factors and PM_2.5_ was analysed using Spearman rank correlation.

#### Land use regression model

Based on the results of PM_2.5_ source apportionment and the common geographically related variables in the LUR model and considering the actual situation in Binzhou, in our study, we selected and obtained a large number of variables, such as road traffic conditions [19], land cover types [20], population density [21], impact of pollution emissions [22], topography [23], soil texture, vegetation indices [24] and large-scale water data. With each monitoring station serving as the central point. Buffered zones ranging from 0.05 to 10.00 km are generated around these monitoring sites. Specifically, for the range of 0.05 to 1.00 km, buffer layers are established at intervals of 0.05 km. In the 1.00 to 2.00 km range, buffer layers are set at intervals of 0.5 km, and for the 2.00 to 10.00 km range, buffer layers are established at intervals of 1.00 km. The area of each type of land use and coverage type, river length, water body area, traffic road length, amount of pollution discharge and elevation, temperature, humidity, WS, BLH, boundary layer dissipation, air pressure, precipitation, vegetation index, landform, terrain relief, distance to the nearest traffic road, distance to the nearest traffic intersection, distance to the nearest water body, soil composition, population density, night light data, and distance to the monitoring site were counted. Taking the PM_2.5_ at the site as the dependent variable, the above geographical variables were selected as the predictor variables and estimated by the random forest regression model. Finally, based on the previously constructed random forest regression model [25]. The 10-fold cross-validation coefficient of determination R^2^ between PM_2.5_ daily estimates and ground-based observations is 0.87, with a RMSE of 17.10 µg/m^3^ (Supplemental Figure S2). Then, points (100 m×100 m) were uniformly distributed in the administrative area. The values of relevant variables at each grid point were collected and subsequently input into random forest model to calculate the estimated PM_2.5_ at each grid point. Kriging interpolation [24, 26] was used to obtain the PM_2.5_ distribution on the surface.

#### Two-stage predictor substitution

The two-stage predictor substitution (2SPS) is a nonlinear extension of the two-stage least squares method and is also completed in two stages. In the first stage, the predicted value of exposure is obtained through nonlinear regression of IVs and exposure, and then the predicted value is substituted for the exposure in the second stage. Specifically, first we fit the following model:

2 $${\varepsilon _1} = f(BL{H_t} + W{S_t}) + {\varepsilon _2}$$

In formula (2), *f* is a nonlinear function, $${BLH}_{t}$$ and $${WS}_{t}$$ are IVs representing the BLH and WS, respectively, and $${\epsilon }_{2}$$ is the model residual. What’s more, BLH and WS may capture some variation of air pollution that is missed by the others, so constructing an IV by combining the two can improve power and avoid the problems of weak IVs [10, 15, 27]. Therefore, we combine the information on BLH and WS on the day of death (lag 0) and the day before death (lag 1) to generate a single pollution-calibrated IV. In the first stage of the 2SPS method in our study, we employ support vector regression (SVR) [28] with a radial kernel to estimate the variations in $${\epsilon }_{1}$$ explained by BLH and WS. The $$\widehat{{\epsilon }_{1}}$$ ( $$\widehat{{\epsilon }_{1}}=$$$$\widehat{\text{E}}\left[{\epsilon }_{1}\right|\text{I}\text{V}\left]\right)$$ is obtained through modelling the changes in $${\epsilon }_{1}$$ using SVR. It’s important to note that $$\widehat{{\epsilon }_{1}}$$ is independent of confounders.

However, the mean number of daily deaths varies over time and can lead to severe overdistribution if left untreated; thus, the time cubic natural spline is included in the model. The mortality rate generally obeys the Poisson distribution, so the function is log (·), and the second-stage regression is shown in formula (3):

3 $$\begin{array}{l}log[E({y_t})] = {\beta _3} + {\beta _4} \cdot {{\hat \varepsilon }_1}\\+ ns\left( {time,df} \right) + {\varepsilon _3}\end{array}$$

In formula (3), ${y}_{t}$ is the number of deaths at time t, ${\beta }_{3}$ is the intercept, ${\beta }_{4}$ is the coefficient of exposure variables estimated by the model, the degrees of freedom for the time spline are 32, $\widehat{{\epsilon }_{1}}$ is the predicted value of ${\epsilon }_{1}$ in formula (2) and ${\epsilon }_{3}$is the random error.

#### Control function

The CFN [9] is another method that uses IVs to solve unobserved confounders. Unlike the conventional IVs method, the CFN method addresses unobserved confounders by incorporating surrogate variables for confounders. The specific procedure of the CFN also comprises two stages.

Similarly, in the first stage, as shown in formula (4), *f* represents a nonlinear function, ${BLH}_{t}$ and ${WS}_{t}$ are IVs representing the BLH and WS, respectively, and ${\epsilon }_{2}$ is the model residual. ${\epsilon }_{2}$ serves as a surrogate variable for confounders. This is because ${\epsilon }_{1}$ is a component of PM_2.5_ and consists of BLH, WS and other factors. These other factors may include confounders, as shown in Fig. 3.

4 $${\varepsilon _1} = f(BL{H_t} + W{S_t}) + {\varepsilon _2}$$

In the second stage regression, ${\epsilon }_{2}$ obtained from the SVR serves as a surrogate variable for confounders, by substituting ${\epsilon }_{2}$ into formula (5), the effect of exposure can be determined [29]. The analysis also accounts for the nonlinear impact of ${\epsilon }_{2}$.

5 $$\begin{array}{l}log\left[ {E\left( {{y_t}} \right)} \right] = {\beta _5} + {\beta _6} \cdot p{m_t}\\+ n{\rm{s}}({\varepsilon _2},df) + ns\left( {time,df} \right) + {\varepsilon _5}\end{array}$$

In formula (5), ${y}_{t}$ represents the number of deaths at time t, ${\beta }_{5}$ is the intercept, ${\beta }_{6}$ is the coefficient of the exposure variables estimated by the model, ${pm}_{t}$ is the PM_2.5_ at time t, the degrees of freedom for the time spline are 32, ${\epsilon }_{2}$ is the residual value of the first-stage regression, and the degrees of freedom for the spline of ${\epsilon }_{2}$are 17, and ${\epsilon }_{5}$ is the random error item of the model.

#### Generalized additive model

To compare with the 2SPS and CFN methods, we employed the general additive model (GAM) to estimate the impact of PM_2.5_ on daily CVDs deaths in our dataset. The model included dummy variables for the day of the week, natural splines for temperature, ozone (O_3_) and time. Similarly, the degrees of freedom for time and temperature were selected through GCV, resulting in 32 degrees of freedom for time, 15 for temperature, and 23 for O_3_. (O_3_ were also obtained from a LUR model based on random forest algorithm, with a 10-fold cross-validation coefficient of determination R^2^ between the O_3_ daily estimate and ground-based observations is 0.90, and the RMSE is 9.09 µg/m^3^. The spatial resolution is 100 m). Specifically, we fit the following model:

6 $$\begin{array}{l}log\left[ {E\left( {{y_t}} \right)} \right] = {\beta _0} + DOW + {\beta _7} \cdot p{m_t} + n{\rm{s}}\left( {te{m_t},df} \right)\\+ ns\left( {time,df} \right) + ns\left( {ozon{e_t},df} \right) + {\varepsilon _6}\end{array}$$

In formula (6), ${y}_{t}$ represents the number of deaths at time t, ${\beta }_{0}$ is the intercept, ${\beta }_{7}$ is the coefficient of PM_2.5_, ${pm}_{t}$ is the PM_2.5_ at time t, ${tem}_{t}$ is the temperature at time *t*, $dow$ is the dummy variable for the day of the week, $time$ represents time, and ${\epsilon }_{6}$ denotes the random error.

#### Time series bootstrap

In addition, when estimating parameter confidence intervals, most previous studies [10, 15, 30] have ignored the autocorrelation of time series data. In our study, the time series bootstrap (tsboot) method [31] was used to estimate the confidence intervals of the parameters. This is because when using the bootstrap method to estimate confidence intervals in a time series study, it’s crucial to address the issue of autocorrelation within the time series data. The tsboot function retains blocks drawn during the sampling process rather than individual samples, preserving correlation information between sequences. Despite the correlation inherent in the time series data, autocorrelation coefficients may be negligible after a certain delay. Therefore, the data is divided into several intervals of fixed length, maintaining the order of sequences while considering the intervals to be approximately independent. Subsequently, bootstrap resampling is conducted on these intervals to estimate parameter confidence intervals. Therefore, our study uses tsboot to find the confidence intervals of parameters.

#### Negative control methods

Furthermore, our study employs negative control methods (NCMs) [32]. By employing the IVs to derive post-outcome variables, we obtain $\widehat{{{\epsilon }_{1}}^{{\prime }}}$ according to formula (2). $\widehat{{{\epsilon }_{1}}^{{\prime }}}$ was used as a negative exposure to examine the presence of unobserved confounders. Through this method, we sought to validate whether unobserved or uncontrolled confounders in our model.

7 $$\begin{array}{l}log\left[ {E\left( {{y_t}} \right)} \right] = {\beta _3} + {\beta _4} \cdot {{\hat \varepsilon }_1} + {\beta _5} \cdot {{\hat \varepsilon '}_1}\\+ ns\left( {time,df} \right) + {\varepsilon _4}\end{array}$$

In formula (7), ${\beta }_{5}$ is the negative exposure coefficient estimated by the model, the degrees of freedom for the time spline are 32, $\widehat{{{\epsilon }_{1}}^{{\prime }}}$ is the predicted value obtained through the IVs after death, and ${\epsilon }_{4}$ is the random error.

#### Software used

All analyses were performed using “geopandas” in Python software (version 3.7.0; Python Software Foundation, 2018) and the “raster”, “gstat”, “randomForest”, “e1071”, “mgcv”, “mda”, “boot” and “splines” packages in R software (version 4.2.1; R Development Core Team, 2016). The effect estimates are presented as excess risks (ERs) and percentage changes with 95% confidence interval (95% CI) for daily mortality associated with a 10 µg/m^3^ increase in PM_2.5_. All the statistical tests were two-tailed, and results with *P* < 0.05 were considered to indicate statistical significance. In the effect estimation model, we employ the Poisson distribution as the distribution family and the logarithmic function as the link function. As a result, we exponentially transform the coefficients and confidence intervals in the model. The main analysis code is shown in Supplemental Code S1.

## Results

### Descriptive statistics of meteorological factors and air pollutants

The average daily temperature was 289.80 ± 1.12 K, and the average WS was 2.47 m/s. The average concentration of PM_2.5_ among those who died of CVDs was 111.63 µg/m^3^, which was much higher than the 75 µg/m^3^ pollutant quality standard for the second-class ambient air functional zone in China’s “Ambient Air Quality Standards” (GB 3095 − 2012). The average number of deaths due to CVDs per day was 41.05, and the highest number of deaths was 72; the average number of deaths due to IHD was 21.59 people per day, and the highest number of deaths was 42; the average number of deaths due to MI was 18.10 people per day, the highest number of deaths due to CVA per day was 12.35, and the highest number of deaths was 29. The average number of daily deaths due to HTN was 1.06, and the highest number of deaths was 8. See Tables 1 and 2 for details.

**Table 1:** Instrumental variables, temperature and PM_2.5_ in Binzhou city ,2016–2020

Variable	Mean	SD	*P*_25_	Median	*P*_75_	Min	Max
WS(m/s)	2.47	1.12	1.84	2.30	2.91	0.27	7.78
BLH(m)	185.38	182.77	55.14	118.05	251.51	12.54	1209.09
Tem/(K)	289.80	9.97	282.50	291.90	298.90	263.20	305.40
PM_2.5_(µg/m^3^)	111.63	8.56	105.08	110.51	117.16	96.85	143.11

Notes: WS is wind speed; BLH is boundary layer height; Tem is temperature; SD is standard deviation; Max is maximum value; Min is minimum value; P_25_ is 25th percentile; P_75_ is 75th percentile.

**Table 2:** Daily deaths from CVDs in Binzhou city,2016–2020

Variable	Mean	SD	*P*_25_	Median	*P*_75_	Min	Max
CVDs	41.05	9.68	34.00	40.50	47.00	15.00	72.00
IHD	21.59	6.13	17.00	21.00	26.00	7.00	42.00
MI	18.10	5.58	14.00	18.00	22.00	6.00	39.00
CVA	12.35	4.31	9.00	12.00	15.00	3.00	29.00
HTN	1.06	1.22	0.00	1.00	2.00	0.00	8.00

Notes: CVDs is cardiovascular diseases; IHD is ischaemic heart disease; MI is myocardial infarction; CVA is cerebrovascular accidents; HTN is hypertension; SD is standard deviation; Max is maximum value; Min is minimum value; P_25_ is 25th percentile; P_75_ is 75th percentile

### Correlation analysis of meteorological factors, PM_2.5_ concentration and temperature

The correlation analysis between BLH, WS and PM_2.5_ showed that meteorological factors were negatively correlated with PM_2.5_ (*r*=-0.17, *r*=-0.19), and there was a strong correlation between PM_2.5_ and meteorological elements, reflecting that BLH and WS are important IVs for studying the impact of air pollutants on human health. Temperature was strongly correlated with BLH (*r* = 0.24) and was also correlated with daily deaths from CVDs(*r*=-0.57). See Table 3 for details.

**Table 3:** Spearman correlation analysis of daily average PM_2.5_, temperature and meteorological parameters

	Tem(K)	BLH(m)	WS(m/s)	PM_2.5_(µg/m^3^)
Tem(K)	1.00			
BLH(m)	0.24	1.00		
WS(m/s)	-0.02	0.48	1.00	
PM_2.5_(µg/m^3^)	-0.71	-0.17	− 0.19	1.00

Notes: WS is wind speed; BLH is boundary layer height; Tem is temperature

### The short-term effect of PM_2.5_ on CVDs mortality

In our IVs method, temperature, short-term fluctuations, and long-term temporal trends explained 62.40% of the average PM_2.5_ variation, and these effects were removed before fitting the predicted values$\widehat{{\epsilon }_{1}}$. IVs explained an average of 29.34% of the remaining variation in PM_2.5_. The predicted value of $\widehat{{\epsilon }_{1}}$ has a correlation of -0.03 with temperature, and $\widehat{{\epsilon }_{1}}$ does not show a temporal trend. The negative exposure control method showed that negative exposure was not associated with CVDs mortality or major CVDs (*P* > 0.05). These findings substantiate the effectiveness of the IV assumptions, demonstrating that in such a context, the established association provides causal estimates for the impact of the locally generated air pollutant PM_2.5_ on daily CVDs mortality rates.

2SPS estimated that the causal relationship between PM_2.5_ (within the day of death and the day before death) and daily CVDs mortality was 1.14% (95% CI: 1.04%, 1.21%) per 10 µg/m^3^ increase, the causal relationship with the mortality rate of IHD mortality was 1.03% (95% CI: 1.02%, 1.19%) for every 10 µg/m^3^ increase, and the causal relationship with MI mortality was 0.95% (95% CI: 0.91%, 1.13%) for every 10 µg/m^3^ increase. The causal relationship with the mortality rate of CVA was 0.88% (95% CI: 0.77%, 1.09%) for every 10 µg/m^3^ increase.

The CFN estimated that the causal relationship between PM_2.5_ (within the day of death and the day before death) and daily CVDs mortality was 1.05% (95% CI: 1.02%, 1.10%) for every 10 µg/m^3^ increase, and the causal relationship with daily IHD mortality was for every 10 µg/m^3^ increase of 1.01% (95% CI: 0.96%, 1.09%). The causal relationship of MI mortality was 0.90% for every 10 µg/m^3^ increase (95% CI: 0.86%, 1.09%). The causal relationship with the mortality rate of CVA was 0.84% (95% CI: 0.71%, 1.01%) for every 10 µg/m^3^ increase.

The GAM estimated that the relationship between PM_2.5_ (within the day of death and the day before death) and daily CVDs mortality was 0.85% (95% CI: 0.77%, 1.05%) for every 10 µg/m^3^ increase, and the causal relationship with daily IHD mortality was 0.63% (95% CI: 0.47%, 0.94%) for every 10 µg/m^3^ increase. The causal relationship of MI mortality was estimated at 0.59% (95% CI: 0.45%, 0.88%) for every 10 µg/m^3^ increase. Similarly, the causal relationship with the mortality rate of CVA was estimated at 0.50% (95% CI: 0.40%, 0.82%) for every 10 µg/m^3^ increase.

However, there was no causal relationship between PM_2.5_ and HTN. See Table 4 for details.

**Table 4:** The analysis of GAM, 2SPS and CFN effects on PM_2.5_ and deaths from main CVDs

Model	CVDs	IHD	MI	CVA	HTN
2SPS	1.14(1.04,1.21)	1.03(1.02,1.19)	0.95(0.91,1.13)	0.88(0.77,1.09)	*P* = 0.43
CFN	1.05(1.01,1.15)	1.01(0.96,1.09)	0.90(0.86,1.09)	0.84(0.71,1.01)	*P* = 0.48
GAM	0.85(0.77,1.05)	0.63(0.47,0.94)	0.59(0.45,0.88)	0.50(0.40,0.82)	*P* = 0.36

Notes: CVDs is cardiovascular diseases; IHD is ischaemic heart disease; MI is myocardial infarction; CVA is cerebrovascular accidents; HTN is hypertension. 2SPS is two-stage predictor substitution method, CFN is control function method, GAM is generalized additive model

## Discussion

This is the first study in China to utilize the IVs method to estimate the impact of PM_2.5_ on CVDs. Employing 2SPS, CFN, GAM, and NCMs, we discovered a significant causal effect of the air pollutant PM_2.5_ on daily mortality related to CVDs, IHD, MI, and CVA. However, no significant association was observed between PM_2.5_ and HTN.

A substantial body of toxicological and human exposure research has revealed the biological pathways connecting PM_2.5_ with daily CVDs in populations. Specifically, studies conducted at relevant doses have identified a significant association between PM_2.5_ exposure and daily mortality rates. Human exposure investigations have demonstrated that exposure to air on busy streets (PM_2.5_=24 µg/m^3^) for 5 h results in a 25% reduction in vascular dilation, an increase in sympathetic nervous system activity, and a decrease in parasympathetic nervous system activity compared to exposure to filtered air (PM_2.5_=3µg/m^3^) [33]. In an intervention experiment, participants who walked on the street for two hours exhibited lower blood pressure when wearing particle-filtering masks than when not wearing masks [34]. Randomized controlled trial focusing on air filtration among elderly individuals revealed improved microvascular function after 48 h of exposure to filtered air [35]. A recent randomized trial involving university students revealed associations between fine particles and HTN, insulin resistance, blood lipids, fasting blood glucose, cortisol, adrenaline, and noradrenaline [36].

In our study, negative exposure demonstrated no discernible impact on mortality rates and did not influence the estimated exposure effects. At the same time, the predicted values of IVs can explain nearly one-third of the remaining changes in PM_2.5_ after controlling for time and temperature. These results demonstrate that the IV assumptions is valid and that this association provides a causal estimate of the effect of the locally produced PM_2.5_ on daily CVDs mortality in such a scenario. Furthermore, our study found that the effect estimates obtained using the IVs method were higher than those obtained using the GAM. Similarly, Schwartz’s study also demonstrated that the effect estimate of a 10 µg/m^3^ increase in PM_2.5_ on daily non-accidental mortality using the IVs method was 1.54% (95% CI: 1.12%∼1.97%), which was significantly higher than the GAM estimate of 0.98% (95% CI: 0.75 ∼ 1.22%) [15]. Recently, Bae applied the IVs method to estimate the effect of O_3_ on population mortality and found that for every 1 ppb increase in O_3_, there was a decrease of 0.37% (95% CI: -0.61%∼-0.14%) in non-accidental mortality. However, in previous linear models, there was no significant association between a 1 ppb increase in O_3_ concentration and daily non-accidental mortality, with a regression coefficient of -0.00024 (*P* = 0.34) [14]. The difference may be attributed to the fact that the GAM only controls for measured confounders to estimate the effect between air pollutants concentration and death from CVDs in the population. There are unobserved confounders that may affect the effect estimate, resulting in smaller results [14]. Another possibility is that the particle variation captured by the IVs primarily consists of elemental and organic carbon particles from local fuel combustion, which may be more toxic than average particles [15].

At the same time, there were differences in the estimated values of the 2SPS and CFN. The estimated value of the 2SPS is higher than that of the CFN. In fact, for the linear model, the estimates of the two methods are equivalent, but for the nonlinear model, studies have shown that the two estimators are different [9]. From the perspective of the model, the CFN, which incorporates surrogate variables of unobserved confounders into the regression, cannot fully control the influence of confounders because the distribution and influence mode of the unobserved confounders are completely unknown. Therefore, when applying the CFN, careful consideration should be given to the application context, acknowledging the unknown distribution and influence patterns of unobserved confounders. Within the causal framework of our study, we assert that the 2SPS yields estimates of the acute effects of the local air pollutant PM_2.5_ on daily CVDs mortality that are more proximate to causal effects. In contrast, the CFN, which relies solely on time spline functions and surrogate variables for unobserved confounders, may not comprehensively account for unobservable factors (this limitation becomes particularly evident when the impact of unobserved confounders on exposure effects is substantial, especially in the presence of multiple unobserved confounding factors in the model). Therefore, unobserved confounders can lead to an underestimation of the short-term effects of air pollution exposure. Additionally, compared to traditional bootstrap methods, the confidence intervals obtained through the tsboot are slightly longer. Therefore, ignoring autocorrelation in time series data may result in an underestimation of the standard errors of effect estimates. (Supplemental Table S3).

In addition, our study has certain limitations. First, the IVs method assumes that there is no association between the IVs and the confounders, which cannot be tested, although our study proved that the 2SPS is not affected by unobserved confounders through the negative exposure method and the previous control, but the possibility of an association between IVs and unobserved confounders remains. Second, our study is based solely on data from one city. The composition and toxicity of PM_2.5_ may vary among different cities, thus the conclusions drawn may not be directly applicable to other cities. Factors such as a city’s specific geographical location, population density, industrial structure, and traffic conditions all influence the generation and dispersion of air pollutants, leading to variations in PM_2.5_ components across cities. To gain a more comprehensive understanding of the impact of PM_2.5_ on CVDs, future research will need to consider expanding the sample scope to encompass data from multiple regions. Additionally, conducting in-depth analyses of regional differences will be necessary to derive more generalizable results.

## Conclusions

Our study used IVs method to estimate the short-term effect of PM_2.5_ exposure on the daily mortality of patients with CVDs (excluding HTN). The analysis was based on the causal framework method, and the observed associations were not subject to unobserved confounders. Additionally, compared to traditional bootstrap methods, the confidence intervals obtained through the tsboot are slightly longer. Compared to GAM, the effect estimates of PM_2.5_ on mortality from CVDs in the city are higher when obtained through the 2SPS and CFN.

### Electronic supplementary material

Below is the link to the electronic supplementary material.

Supplementary Material 1

## Data Availability

Data are provided in the supplementary information files, specifically in Supplemental Table S1. The analytical code is presented in the Supplemental Code S1.

## Abbreviations

CVDs: Cardiovascular diseases
2SPS: Two-stage predictor substitution
CFN: Control function GAM: Generalized additive model
IVs: Instrumental variables
LUR: Land use regression
SVR: Support vector regression
ICD-10: International Statistical Classification of Diseases and Related Health Problems 10th Revision
O_3_: Ozone
IHD: Ischaemic heart disease
CVA: Cerebrovascular accident
MI: Myocardial infarction
HTN: hypertension
WS: Wind speed
BLH: Boundary layer height
NCMs: Negative control methods
ERs: Excess risks
CI: Confidence interval
SD: Standard deviation
