# Supplementary material for: Estimating the short-term effect of PM2.5 on the mortality of cardiovascular diseases based on instrumental variables
Source: BMC Public Health. 2024 Aug 1;24:2085. doi: 10.1186/s12889-024-18750-0 (PMC11295497; doi:10.1186/s12889-024-18750-0)
Supplement: Supplementary file 1 — Supplementary Material 1 [file 12889_2024_18750_MOESM1_ESM.docx]

**SUPPLEMENTARY MATERIALS**

There are 3 tables, 2 Figures and 1 analysis code in our supplementary materials.

**Table of Contents Page**

[Supplemental Figure S1 2](#_Toc11330)

[Supplemental Table S1 3](#_Toc25424)

[Supplemental Table S2 4](#_Toc32536)

[Supplemental Figure S2 8](#_Toc30450)

[Supplemental Table S3 9](#_Toc30450)

[Supplemental Code S1 10](#_Toc30450)


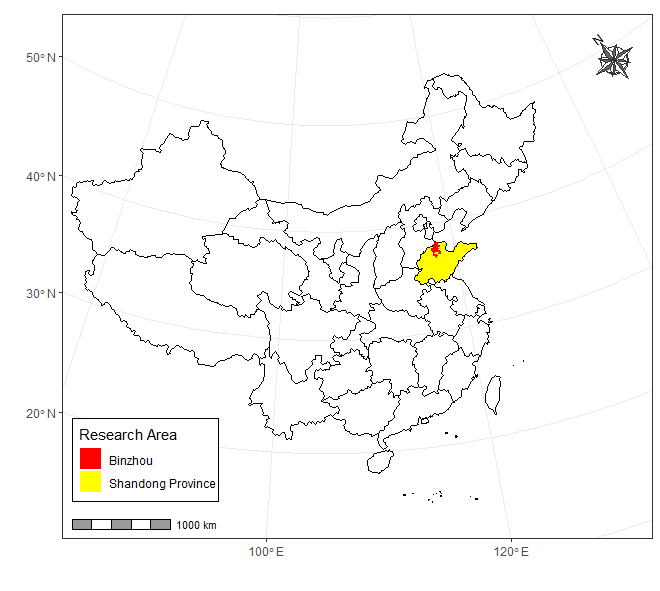


**Supplemental Figure S1** Location of the study area in China

**Supplemental Table S1** Estimation of PM_2.5_ random forest model variables and sources

| Variable | Source |
| --- | --- |
| Population density | Raster data of global population distribution (https://landscan.ornl.gov) |
| Night lights | Resource Science and Data Center of Chinese Academy of Sciences (https://www.resdc.cn/) |
| The Traffic | Openstreetmap（http://www.openstreetmap.org/） |
| Land Cover Type | Zenodo（https://zenodo.org/record/5210928） |
| Pollutant Emission | Gaode Map API (https://lbs.amap.com/) |
|  | Binzhou Ecological Environment Bureau (http://hb.binzhou.gov.cn/) |
|  | Zibo Ecological Environment Bureau (http://epb.zibo.gov.cn/) |
|  | Jinan Ecological Environment Bureau (http://jnepb.jinan.gov.cn/) |
|  | Cangzhou Ecological Environment Bureau (http://hb.cangzhou.gov.cn/) |
|  | Dezhou Ecological Environment Bureau (http://dzbee.dezhou.gov.cn/) |
|  | Dongying Ecological Environment Bureau (http://sthj.dongying.gov.cn/) |
| The Waters | Openstreetmap（http://www.openstreetmap.org/） |
| Vegetation Index | China Science and Technology Resource Sharing Network (https://www.escience.org.cn/) |
| Meteorological | European Meteorological Center (https://www.ecmwf.int/) |
| Terrain | Resource Science and Data Center of Chinese Academy of Sciences (https://www.resdc.cn/), |
| Elevation | Geospatial Cloud (https://www.gscloud.cn/) |
| Soil Texture | Geospatial Cloud (https://www.gscloud.cn/) |
| PM_2.5_ and Monitoring Stations | World Air Quality Index Network (http://www.waqi.info/cn/) |

**Supplemental Table S2** List of main variables

| Category | Variable Name | Buffer Zone | Resolution | Unit |
| --- | --- | --- | --- | --- |
| road length | Distance to nearest railway | 50m、100m、150m、200m、250m、300m、350m、400m、500m、550m、600m、650m、700m、750m、800m、850m、900m、950m、1000m、1500m、2000m、3000m、4000m、5000m、  6000m、  7000m、  8000m、  9000m、  10000m |  | m |
|  | Distance to nearest highway |  |  |  |
|  | Distance to main road |  |  |  |
|  | Distance to nearest common street |  |  |  |
|  | Distance to nearest alley |  |  |  |
|  | Distance to nearest minor road |  |  |  |
|  | Distance to nearest major road |  |  |  |
|  | Distance to the road to the nearest residential area |  |  |  |
|  | Road/entrance to nearest expressway/expressway |  |  |  |
|  | Distance to connecting roads connecting major roads with other Main road |  |  |  |
|  | Closest distance to minor road intersection |  |  |  |
|  | Closest distance to a common road junction |  |  |  |
|  | Closest distance to arterial road fork |  |  |  |
|  | All roads |  |  |  |
|  | Highway |  |  |  |
|  | Trunk road |  |  |  |
|  | Secondary road |  |  |  |
| **Continued Supplemental Table S2** List of main variables | | | | |
| Category | Variable Name | Buffer Zone | Resolution | Unit |
| Vegetation Index | NDVI | - | 1km | —— |
| Population Density | Population | - | 1km | people/km^2^ |
| Meteorological data | Boundary Layer Height | - | 0.01° | m |
|  | 2m dew point temperature |  |  | K |
|  | 2m air temperature |  |  | K |
|  | Precipitation |  |  | mm |
|  | Surface pressure |  |  | 10^6^ Pa |
|  | North-south wind speed |  |  | m/s |
|  | East-west wind speed |  |  | m/s |
|  | Relative humidity |  |  | % |
|  | Boundary layer dissipation |  |  | Jm^2^ |
| Elevation | Elevation | - | 1km | m |
| night lights | Night lights | - | 0.04° | - |
| **Continued Supplemental Table S2** List of main variables | | | | |
| Category | Variable Name | Buffer Zone | Resolution | Unit |
| Land Cover Type | Farmland | 50m、100m、150m、200m、250m、300m、350m、400m、500m、550m、600m、650m、700m、750m、800m、850m、900m、950m、1000m、1500m、2000m、3000m、4000m、5000m、  6000m、  7000m、  8000m、  9000m、  10000m | 30m | m^2^ |
|  | Grassland |  |  |  |
|  | Bush |  |  |  |
|  | Wetlands |  |  |  |
|  | The Waters |  |  |  |
|  | Naked |  |  |  |
|  | Impermeable layer |  |  |  |
|  | Forest |  |  |  |
| Pollution discharge point | Gas station | - | - | - |
|  | Thermal power plant |  |  |  |
|  | Comprehensive market |  |  |  |
|  | Shopping mall |  |  |  |
|  | Restaurant |  |  |  |
|  | Bus stop |  |  |  |
| **Continued Supplemental Table S2** List of main variables | | | | |
| Category | Variable Name | Variable Name | Resolution | Unit |
| Water | Channel length | 50m、100m、150m、200m、250m、300m、350m、400m、500m、550m、600m、650m、700m、750m、800m、850m、900m、950m、1000m、1500m、2000m、3000m、4000m、5000m、  6000m、  7000m、  8000m、  9000m、  10000m | - | m |
|  | Distance to nearest river |  |  | m |
|  | Distance to nearest dam |  |  | m |
|  | Distance to nearest stream |  |  | m |
|  | Water area |  |  | m^2^ |
| Terrain | Terrain | - | - | - |
| Soil Texture | Soil Texture | - | 1km | - |
| PM_2.5_ | PM_2.5_ | - | *-* | *μg*/*m*^3^ |


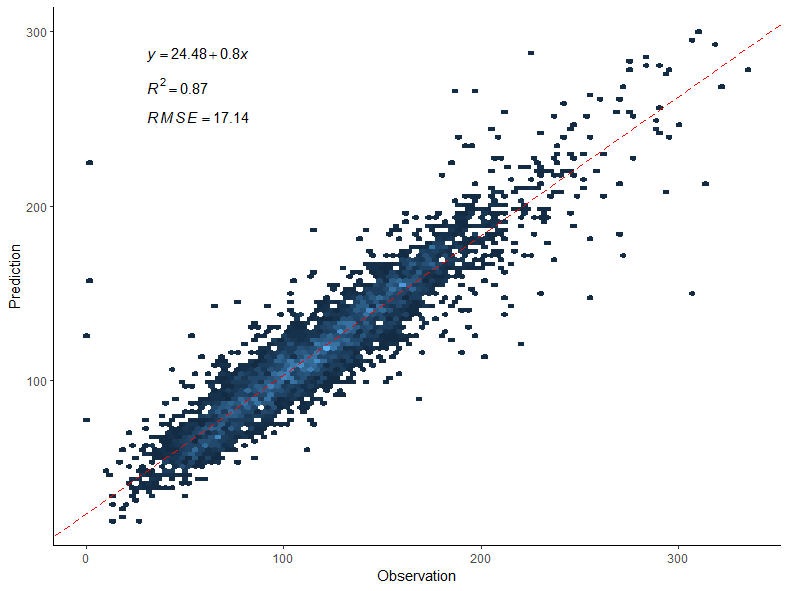


**Supplemental Figure S2** Random forest model prediction and actual cross-validation results

**Supplemental Table S3** Comparison between the tsboot and bootstrap intervals

|  | 2SPS(tsboot) | CF(tsboot) | 2SPS(bootstrap) | CF(bootstrap) |
| --- | --- | --- | --- | --- |
| CVD | 1.14(1.04,1.21) | 1.05(1.01,1.15) | 1.14(1.02,1.19) | 1.05(1.00,1.13) |
| IHD | 1.03(1.02,1.19) | 1.01(0.96,1.09) | 1.03(0.99,1.15) | 1.01(0.89,1.07) |
| MI | 0.95(0.91,1.13) | 0.90(0.86,1.09) | 0.95(0.89,1.10) | 0.90(0.83,1.04) |
| CVA | 0.88(0.77,1.09) | 0.84(0.71,1.01) | 0.88(0.74,0.98) | 0.84(0.70,0.96) |
| HTN | P=0.43 | P=0.48 | P=0.43 | P=0.48 |

**Supplemental Code S1** Analytic code

# The required R package needs to be installed before analysis,You can copy the following code.

# install.packages(c("mgcv", "splines","e1071","mda","boot","pacman"))

library("pacman")

p_load(mgcv, splines,e1071,mda,boot)

# Step1:

# Data includes temperature, daily death toll, PM_2.5_ concentration, time variable, boundary layer height and wind speed.

# pm2.5: The concentration of pm2.5 in a numeric format, eg. 150.

# time:The time in a Date format, eg. 2016-01-01.

# dow: The day of week variable in a factor format, eg. Monday.

# temperature: The temperature in a numeric format, eg. 12.

# optimal.df :The degrees of freedom of natural spline function with time variable for predicting the exposure PM_2.5_ in a numeric format, eg. 15.

# resid1:The residual change of pm2.5 after controlling the confounding factors of temperature and time trend.

pm2.5<-data$pm2.5

time<-unclass(data$time)

use <- complete.cases(pm2.5, time)

br.fit<- bruto(time, pm2.5)

optimal.df<- br.fit$df

model1<-gam(pm2.5~ns(time,df=optimal.df)+

as.factor(dow)+

s(temperature,bs="ad"),

data=data,family=gaussian)

resid1<-residuals(model1)

data$resid1<-resid1

# Step2: Two-stage predictor substitution

# Data includes temperature, daily death toll, PM2.5 concentration, time variable, boundary layer height and wind speed.

# pm2.5: The concentration of pm2.5 in a numeric format, eg. 150.

# time :The time in a Date format, eg. 2016-01-01.

# dow: The day of week variable in a factor format, eg. Monday.

# temperature: The temperature in a numeric format, eg. 12.

# optimal.df :The degrees of freedom of natural spline function with time variable for predicting the exposure pm2.5 in a numeric format, eg. 15.

# resid1:The residual change of pm2.5 after controlling the confounding factors of temperature and time trend.

# optimal.df1: The degrees of freedom of natural spline function with time variable for predicting the deaths in a numeric format, eg. 15.

# optimal.df2: The degrees of freedom of natural spline function with temperature for predicting the deaths in a numeric format, eg. 15.

# ppm2.5: The predicted value of pm2.5.

# pbl:The height of the planetary boundary layer in a numeric format, eg. 302.

# ws:The wind speed in a numeric format, eg. 0.8.

# deaths: The daily death toll in a numeric format, eg. 40.

# Return the health effect of pm2.5 and its 95% CI by bootstrapping of time series.

model2<-svm(resid1~pbl+ws,data=data,kernel='radial')

ppm2.5<-predict(model2)

data$ppm2.5<-ppm2.5

deaths<-data$deaths

time<-unclass(data$time)

use <- complete.cases(deaths, time)

br.fit<- bruto(time[use], deaths[use])

optimal.df1<- br.fit$df

temperature<-unclass(data$temperature)

use <- complete.cases(deaths,temperature)

br.fit<- bruto(temperature[use],deaths[use])

optimal.df2<- br.fit$df

model3<-gam(deaths~ppm2.5+

ns(time,df=optimal.df1)+

as.factor(dow)+

ns(temperature,df=optimal.df2),

family=quasipoisson,data=data)

summary(model3)

bootf<-function(data)

{mod<-gam(deaths~ppm2.5+

ns(time,df=optimal.df1)+

as.factor(dow)+

ns(temperature,df=optimal.df2),

family=quasipoisson,data=data)

return(mod$coef[2])

}

boot3<-tsboot(data,bootf, R=2000, l=20, sim ="fixed")

quantile(boot3$t[,1], probs = c(0.025,0.975))

# Step3: Control function

# Data includes temperature, daily death toll, PM2.5 concentration, time variable, boundary layer height and wind speed.

# time :The time in a Date format, eg. 2016-01-01.

# dow: The day of week variable in a factor format, eg. Monday.

# temperature: The temperature in a numeric format, eg. 12.

# resid1: The residual change of pm2.5 after controlling the confounding factors of temperature and time trend.

# optimal.df1: The degrees of freedom of natural spline function with time variable for predicting the deaths in a numeric format, eg. 15.

# optimal.df2: The degrees of freedom of natural spline function with temperature or predicting the deaths in a numeric format, eg. 15.

# resid2:The residual resid1 and the instrument variables are obtained for support vector regression.

# pbl:The height of the planetary boundary layer in a numeric format, eg. 302.

# ws:The wind speed in a numeric format, eg. 0.8.

# deaths:The daily death toll in a numeric format, eg. 40.

# Return the health effect of pm2.5 and its 95% CI by bootstrapping of time series.

model4<-svm(resid1~pbl+ws,data=data,kernel='radial')

resid2<-residuals(model4)

data$resid2<-resid2

deaths<-data$deaths

time<-unclass(data$time)

use <- complete.cases(deaths, time)

br.fit<- bruto(time[use], deaths[use])

optimal.df1<- br.fit$df

temperature<-unclass(data$temperature)

use <- complete.cases(deaths,temperature)

br.fit<- bruto(temperature[use], deaths[use])

optimal.df2<- br.fit$df

model5<-gam(deaths~pm2.5+resid2+

ns(time,df=optimal.df1)+

as.factor(dow)+

ns(temperature,df=optimal.df2),

family=quasipoisson,data=data)

summary(model5)

bootf<-function(data)

{mod<-gam(deaths~pm2.5+resid2+

ns(time,df=optimal.df1)+

as.factor(dow)+

ns(temperature,df=optimal.df2),

family=quasipoisson,data=data)

return(mod$coef[2])

}

boot4<-tsboot(data,bootf, R=2000, l=20, sim ="fixed")

quantile(boot4$t[,1], probs = c(0.025,0.975))

# Step4: Negative exposure

# You can do the same process as step 2 to test the instrumental variables assumption, with the exposure becoming the exposure after the outcome ( negative exposure) .
